# An evidence base for Zhang-fu acupuncture and polyherbal formulations in supportive care of people with cancer

**DOI:** 10.2471/BLT.25.293714

**Published:** 2025-10-13

**Authors:** Vu Thi Thu Trang, Le Thi Thuy, Lai Thanh Hien, Tran Thi Hai Van, Le Van Truong

**Affiliations:** a National Hospital of Traditional Medicine, Hanoi, Viet Nam.; b Traditional Medicine Hospital, Ministry of Public Security, 278 Luong The Vinh, Dai Mo, Hanoi 100000, Viet Nam.; c Hanoi Medical University, Hanoi, Viet Nam.

According to World Health Organization (WHO) estimates, the global cancer incidence in 2022 was 20 million cases, a number that is projected to increase to 35 million by 2050.^1^ Despite therapeutic advances, cancer places a substantial burden on patients’ health and quality of life. Traditional, complementary and integrative medicine, as defined in the new WHO *Global traditional medicine strategy 2025–2034*,^2^ has the potential to mitigate toxicities associated with biomedical treatments and to support patients in advanced stages of the disease. 

Traditional medicine focuses on restoring physiological balance and enhancing resilience, contrasting with the reductionist approach of biomedical therapies.^3^^,^^4^ In clinical practice, the application of traditional medicine in supporting cancer treatment predominantly employs a symptomatic approach, identifying primary patient symptoms as clinical syndromes. In cancer supportive care, the focus is on interventions to mitigate symptoms and treatment-related toxicities such as fatigue, nausea, pain and myelosuppression.^5^ These syndromes are then correlated with descriptions in classical, conventional medical texts to determine appropriate therapeutic principles and medicinal plant formulas.^5^ In cancer care, symptomatic treatment approaches commonly focus on pain management, which has been shown to be effective based on immunological markers.^6^^,^^7^ Nevertheless, this strategy often results in heterogeneous syndrome classifications and corresponding prescriptions, thereby demanding considerable expertise from traditional medicine practitioners, while still lacking robust clinical evidence.^5^

Alternatively, a biomedical approach establishes syndrome differentiation on the basis of the specific cancer type.^4^ However, using biomedical diagnoses as the starting point for applying traditional medicine can lead to inconsistencies between biomedical and traditional disease models, affecting treatment decisions. For example, patients diagnosed with liver cancer using biomedical techniques in the early stages of the disease often exhibit no symptoms. However, based on traditional medicine, they may be described as having the liver becoming as hard as stone, a manifestation associated with late-stage disease. Based on this classification, patients may be prescribed treatments intended for late-stage cancer instead of treatments appropriate for their early-stage condition, leading to inappropriate medication use.

Both approaches face a common challenge of fragmented diagnoses, which complicates treatment selection. For example, liver and pancreatic cancers can be classified by traditional medicine into eight and nine syndromes, respectively.^5^ Such heterogeneity in diagnostic and therapeutic practices hampers the development of standardized criteria and treatment protocols, particularly in defining research inclusion and exclusion criteria.^8^

The Zang-Fu theory, central to traditional Chinese medicine, holds that disease arises from yin–yang imbalances within the organ systems. The theory views the body as an interconnected system, where balanced organ function, yin–yang, and *qi* and *xue *circulation maintain health. The theory highlights the dynamic interaction between the five Zang and their corresponding Fu organs, with diagnostic methods identifying disharmonies such as deficiency (*xu*) or excess (*shi*).^9^^,^^10^ Dysfunction in one or more organs produces observable clinical signs, which physicians use to identify underlying causes and guide interventions to restore balance. Rather than focusing only on symptoms or biomedical mechanisms, treatments aim to restore yin–yang balance through personalized approaches such as acupuncture or medicinal plant formulations.^3^^,^^6^

The Zang-Fu framework provides a unifying lens to address fragmented diagnostic practices. By linking clinical manifestations to organ-based imbalances rather than isolated symptoms or biomedical categories, the framework enables standardized, clinically applicable diagnostic criteria. This approach supports consistent therapeutic protocols with acupuncture or medicinal plant formulas systematically derived from identified patterns, reducing heterogeneity and laying the foundation for reproducible, evidence-based supportive care in cancer.

## Contemporary oncology gains and limits 

Contemporary oncology has undergone a transformative evolution, propelled by innovations in cytotoxic chemotherapy, radiotherapy, molecularly targeted therapies, immunotherapy and genomic-guided personalized medicine. Chemotherapy and radiotherapy disrupt neoplastic proliferation through broad cytotoxic mechanisms, while targeted therapies exploit tumour-specific molecular aberrations for greater precision. Immunotherapy harnesses adaptive immunity to achieve durable responses in select patient cohorts, and personalized medicine tailors interventions to individual tumour genomics, minimizing off-target effects. These advances have markedly extended survival and improved clinical outcomes across diverse cancer types.^11^

Nevertheless, significant limitations persist. The systemic toxicities of chemotherapy, manifested through non-specific effects such as nausea, myelosuppression and neurocognitive decline, not only erode patients’ functional capacity but also profoundly disrupt the body’s intrinsic physiological balance. These disturbances accumulate over time, leading to progressive debilitation and making it increasingly difficult for patients to sustain their quality of life. In parallel, therapeutic resistance, driven by tumour heterogeneity, continues to undermine long-term treatment efficacy. 

In many low- and middle-income countries, the prohibitive cost and infrastructural demands of these modalities restrict access, resulting in disproportionately high cancer mortality rates. Moreover, the dominant focus of modern oncology on tumour eradication often overlooks the essential need for cancer supportive care, particularly in advanced disease stages, where restoring balance and preserving quality of life becomes paramount.^12^

## Challenges in integration

Efforts to integrate Zang-Fu theory with modern oncology by aligning its patterns with biomedical pathologies have led to inconsistencies between the descriptions of modern medicine and traditional medicine, resulting in heterogeneous treatment approaches and a loss of confidence in the effectiveness of traditional medicine. For instance, cancer-related dyspnea may be attributed to lung *qi* deficiency, kidney yang depletion or phlegm-damp accumulation, resulting in overlapping syndromes that compromise diagnostic reliability and therapeutic consistency, as evidenced in narrative reviews of traditional Chinese medicine in oncology.^5^ Standardizing protocols for cancer diagnosis and treatment remains challenging, leading to treatment plans that vary widely among experts due to subjective interpretations.^3^ This overlap with biomedical symptom management, such as fatigue treated by both Zang-Fu tonics and pharmaceuticals, generates redundancy rather than synergy, diminishing the distinct systemic contributions of traditional medicine. Research faces similar obstacles: heterogeneous classifications hinder meta-analyses, and the lack of validated diagnostic tools impedes randomized controlled trials (RCTs).

## Prospective research agenda

To translate Zang-Fu theory in cancer supportive care and address outlined challenges, we propose a multiphase research agenda. First, develop a diagnostic instrument, through a structured questionnaire based on traditional Chinese medicine’s diagnostic tetrad, drawing upon symptoms documented in relation to Zang-Fu theory.^3^ Second, implement syndrome delineation via cluster analysis by employing sibling cluster analysis, such as the Lantern tree method.^13^ This method offers a theoretically grounded approach to identify distinct Zang-Fu typologies and excels at detecting meaningful clusters, distinguishing deficiency from excess states more effectively than conventional techniques. Third, validate an objective tool, through synthesizing cluster findings into a standardized diagnostic algorithm, minimizing interpractitioner variability and enabling scalability. Finally, conduct clinical trials for therapeutic efficacy, building on the validated characteristics of the identified syndromes, and drawing on both classical and contemporary medical literature to develop acupoint prescriptions or medicinal plant formulations aimed at restoring yin–yang balance within the Zang-Fu system. These formulations will subsequently be tested in RCTs, thereby generating robust scientific evidence of their therapeutic efficacy in cancer supportive care. 

Our approach identifies traditional clinical patterns in cancer patients through the lens of Zang-Fu theory and supports the development of diagnostic tools, standardized inclusion and exclusion criteria, and consistent therapeutic protocols. Rather than only prioritizing direct antitumour effects, this framework emphasizes restoring yin–yang balance, enhancing the body’s self-regulatory capacity and strengthening systemic resilience, thereby improving treatment consistency and overall patient outcomes.

## Integrating traditional medicine

Current integrative approaches are largely limited to acupuncture and polyherbal formulations aimed at alleviating chemotherapy- or radiotherapy-induced symptoms such as fatigue, nausea, pain and myelosuppression.^5^ However, the current integrative approach applications are inconsistent, varying across practitioners, facilities and regions, which complicates outcome assessment.^4^^,^^6^^,^^8^

The Zang-Fu theory offers a consistent framework by mapping clinical manifestations onto specific organ imbalances. Interventions, including acupuncture and medicinal plant formulations, can thus be tailored to identified patterns, creating standardized diagnostic and treatment protocols.^9^^,^^10^ A yin–yang rebalancing approach within this framework could complement modern treatments across all cancer stages, enhancing patient resilience and quality of life.^10^

Globally, traditional medicine integration varies widely. In Hong Kong Special Administrative Region, China, and Viet Nam, hospitals have implemented fully integrated oncology programmes, while in many other countries, integration is limited, early detection is rare and high treatment costs restrict access.^12^ In these contexts, low-cost, accessible interventions could provide feasible supportive care, alleviate symptoms and improve quality of life.

While the Zang-Fu theory offers a transformative approach to cancer supportive care by addressing systemic imbalances overlooked in tumour-centric oncology, its integration is hindered by diagnostic fragmentation and lack of standardization. Overcoming these challenges requires structured research, including development of diagnostic tools, syndrome identification, therapy standardization and clinical trials, to build an evidence base, guide policy and support safe, scalable integration of traditional medicine. This research is especially critical for low- and middle-income countries, where early detection and treatment access remain constrained. The proposed research agenda aims to bridge this gap. 

By providing a coherent framework for standardizing diagnostic practices and guiding individualized, organ-based interventions, such as acupuncture and medicinal plant formulations, the Zang-Fu theory can enable restoration of yin–yang balance and help reduce heterogeneity in cancer care. Integrating traditional holistic principles with scientific rigour can promote evidence-based, patient-centred, and globally applicable supportive care, ultimately enhancing outcomes and improving equitable access.
